# Memory as Social Glue: Close Interpersonal Relationships in Amnesic Patients

**DOI:** 10.3389/fpsyg.2012.00531

**Published:** 2012-12-04

**Authors:** Patrick S. R. Davidson, Héloïse Drouin, Donna Kwan, Morris Moscovitch, R. Shayna Rosenbaum

**Affiliations:** ^1^School of Psychology, University of OttawaOttawa, ON, Canada; ^2^Bruyère Research Institute, University of OttawaOttawa, ON, Canada; ^3^Centre for Stroke Recovery, Heart and Stroke Foundation of OntarioToronto, ON, Canada; ^4^Department of Psychology, York UniversityToronto, ON, Canada; ^5^Department of Psychology, University of TorontoToronto, ON, Canada; ^6^Rotman Research Institute, BaycrestToronto, ON, Canada

**Keywords:** amnesia, memory, hippocampus, medial temporal lobe, friendship, social networks

## Abstract

Memory may be crucial for establishing and/or maintaining social bonds. Using the National Social life, Health, and Aging Project questionnaire, we examined close interpersonal relationships in three amnesic people: K.C. and D.A. (who are adult-onset cases) and H.C. (who has developmental amnesia). All three patients were less involved than demographically matched controls with neighbors and religious and community groups. A higher-than-normal percentage of the adult-onset (K.C. and D.A.) cases’ close relationships were with family members, and they had made few new close friends in the decades since the onset of their amnesia. On the other hand, the patient with developmental amnesia (H.C.) had forged a couple of close relationships, including one with her fiancé. Social networks appear to be winnowed, but not obliterated, by amnesia. The obvious explanation for the patients’ reduced social functioning stems from their memory impairment, but we discuss other potentially important factors for future study.

## Introduction

What is memory for? Episodic memory enables one to capture the precise details of an experience, and then to recollect this information rapidly whenever and wherever needed. Typical examples of its evolutionary value focus on the individual navigating the world, but the advantages conferred by episodic memory may be more far-reaching than often appreciated. For example, interactions with other people are important for our survival and wellbeing (Eisenberger and Cole, 2012). Might episodic memory be crucial for establishing and/or maintaining interpersonal relationships?

To address this question, we examined social relationships in three amnesic patients. In such patients, damage to medial temporal, diencephalic, and/or basal forebrain structures yields a profound and relatively specific impairment in episodic memory, with other faculties (i.e., sensory-perceptual, cognitive, and motor) remaining essentially intact (for recent reviews, see Squire and Wixted, 2011; Rosenbaum et al., 2012). On the one hand, many of the psychological functions necessary for interpersonal relationships are preserved in amnesia. For example, amnesic patients usually are able to hold a simple conversation, as long as they are not distracted. Their semantic knowledge about the basic rules of social interaction and friendship is intact; many retain exemplary social graces (Corkin, 1984; Rosenbaum et al., 2005). Many also retain considerable semantic knowledge about their family members and friends from their pre-morbid lives. Amnesic patients are even capable of learning whether new people are associated with positive versus negative outcomes (e.g., Tranel and Damasio, 1993; Claparède, 1995; Turnbull and Evans, 2006), and often seem to grow more comfortable with people and objects following repeated exposure to them (Johnson et al., 1985). Their ability to infer other people’s thoughts, feelings, and intentions is normal, at least under many circumstances (Rabin et al., in press; Rosenbaum et al., 2007).

On the other hand, several psychological functions that would appear to be crucial for social bonding are impaired in amnesic patients. The most obvious is episodic memory itself: the ability to consciously recollect experiences (including the often arbitrary associations among their elements, such as people, names, places, times, and things) and to use these memories flexibly and update them when necessary (Rosenbaum et al., 2012). Thus, amnesic patients often fail to learn who new people are, even after hundreds of encounters. Even when they can accrue bits of information, they may be unable to bind these together into coherent entities, instead retrieving only fragments of previous events. In addition, difficulty updating their memories means that even when they *can* remember something about old friends’ and family members’ lives, they may fail to keep track of changes and consequently commit social faux pas errors (e.g., Patient: *How’s your father?* Friend: *Remember – He died last year*; Klein et al., 2009). Amnesic patients’ inability to discuss current events (even momentous ones; Davidson et al., 2005; Ogden, 2005; Rosenbaum et al., 2005) and to share warm reminiscences with others may hamper social bonding (Alea and Bluck, 2003). Episodic memory (and the medial temporal lobe memory system) also appears to support other potentially important abilities, including aspects of discourse (Duff and Brown-Schmidt, 2012), thinking about one’s future to help plan social interactions (Rosenbaum et al., 2005; Hassabis et al., 2007; Squire et al., 2010), empathy (Ciaramelli et al., submitted), and social problem-solving (Sheldon et al., 2011). For these reasons, amnesic patients have been described as ending up “interactionally marooned”^1^ (Ogden, 2005; Duff et al., 2009). In converging evidence from healthy people, individual differences in social network size are predicted by episodic memory (among other cognitive functions; Stiller and Dunbar, 2007; Dunbar, 2008).

Although there already exists a literature on the negative consequences of brain injuries and dementia for social functioning (e.g., Finset et al., 1995; Morton and Wehman, 1995; Dijkers, 2004; Andrew et al., 2011; Henry et al., 2012), relatively few of these studies have examined the potential influence of memory impairment *per se*, and, regardless, these patients often have other important cognitive and behavioral problems, including disorganized thinking and behavior, emotional lability, poor inhibition, poor insight, anxiety, and depression. As far as we are aware, there exist only a few reports on social functioning in amnesia. The most recent one was surprising: Duff et al. (2008) described Angie, a densely amnesic woman who established several new close interpersonal relationships post-injury, including a new marriage (see also Wilson, 1999)! This report seemed at odds with our anecdotal observations of other patients, as well as with other case reports (Kaushall et al., 1981; Tate, 2002; Warren et al., 2012). The existing literature, however, is based primarily on clinical observations and qualitative descriptions. Thus, to examine close friendships in amnesia in a more systematic way, we administered a formal questionnaire.

## Materials and Methods

### Participants

*Patient K.C*. is a 60-year-old right-handed man with 14 years of education. He sustained a head injury in a motorcycle accident in 1981 (for more detail, see Rosenbaum et al., 2005). His brain lesions are widespread; most notable is near-complete loss of the hippocampus, septal area, and posterior thalamus bilaterally, and damage to the parahippocampal cortices, amygdala, mammillary bodies, and anterior thalamus that is greater in the left than right hemisphere. Despite the extent of K.C.’s brain damage and resulting anterograde amnesia, his retrograde amnesia is relatively limited to episodic memory, affecting his ability to re-experience, and imagine personal events from across his lifetime (Rosenbaum et al., 2009). In contrast, K.C.’s semantic memory for personal and world facts learned prior to his head injury is relatively spared (Westmacott et al., 2001). His overall intellectual ability, language, visual perception, short-term and working memory, and executive function are also intact, though he exhibits some psychomotor slowing that may account for low average verbal fluency.

With respect to personality and social function, K.C. is very polite, cooperative, and friendly, though somewhat tranquil and reserved. The latter characteristic represents a change from his pre-morbid personality, which he and others describe as thrill-seeking and extroverted. He rarely initiates conversation but otherwise interacts well with others. Formal testing of K.C.’s theory of mind indicates that he is able to take other people’s perspectives and infer their thoughts and feelings without difficulty (Rosenbaum et al., 2007).

*Patient D.A*. is a 60-year-old right-handed man with 17 years of education. He contracted herpes encephalitis in 1993, which produced a pattern of volume loss most prominent in the anterior and medial temporal lobe, including the hippocampus, amygdala, and parahippocampal cortex, and orbitofrontal cortex bilaterally, with overall more extensive damage in the right hemisphere (shown in Rosenbaum et al., 2008). D.A.’s ensuing memory problems required him to leave his professional career. Neuropsychological assessment revealed marked anterograde amnesia but intact performance on tests of intellectual function, language, visual perception, short-term and working memory, and executive function. A temporal gradient in remote autobiographical memory and semantic memory was also characterized, with poorer performance for events experienced in the 30 years prior to his injury (Rosenbaum et al., 2008), and for names and words that became well known in the 5 years prior to his injury (Westmacott and Moscovitch, 2002).

D.A. behaves appropriately in a social context and values his relationships with his wife, children, and friends. He has retained a warm-hearted, amicable, and gregarious personality, with a keen sense of humor that he uses to compensate for his memory loss.

*Patient H.C*. is a 23-year-old right-handed woman with developmental amnesia resulting from a probable hypoxic event at the age of 1 week. MRI has revealed significant bilateral reduction in hippocampal volumes, with the remaining tissue at least 2 SD smaller than that of age-matched healthy controls. When H.C. was included in a group of developmental amnesic patients, the group showed additional reduced volumes in the thalamus and basal ganglia bilaterally and in retrosplenial cortex in the right hemisphere (see Vargha-Khadem et al., 2003, patient E6, for additional neuroanatomical details).

H.C.’s cognitive profile features average IQ and normal fluency and semantic memory. She completed regular-stream high school and a year of community college. In contrast to her generally preserved cognitive functioning, H.C. has not developed normal episodic memory, based on reports from family members and as seen on clinical and experimental measures (Vargha-Khadem et al., 2003; Kwan et al., 2010). Her episodic memory impairment extends to memory for public events (Rosenbaum et al., 2011) and to imagination of future experiences in response to cue words (Kwan et al., 2010) but not to cues that are more elaborative/detailed or that depict commonplace scenes (Hurley et al., 2011; see Rosenbaum et al., 2011 for detailed neuropsychological profile).

H.C. has a bubbly, agreeable, and generally positive disposition. She is aware of having a memory problem, but approaches her impairment with light-hearted humor and acceptance. She is even-tempered, friendly, and talkative, often telling jokes and stories with great animation and detail in between testing (although these stories are typically the same as she has told in the past). When alone, H.C. passes the time by reading fiction, watching television, and browsing online social media/sharing sites such as Facebook and Pinterest.

Demographic and neuropsychological data for the patients are presented in Table 1.

**Table 1:** **Neuropsychological profiles of the amnesic patients**.

	K.C.	D.A.	H.C.
**WAIS-R (STANDARD SCORE)**			
FSIQ	88 (99)^1^	117	106
VIQ	96 (99)	121	104
PIQ	79 (99)	106	106
AM-NART (standard score)	102	117	101.28
WAIS-R vocab (scaled score)	9	12	–
Boston naming (/60)	57	56	58
Semantic fluency^2^ (scaled score)	10	12	>14
**WMS-R**			
General memory (standard score)	61	74	49*
Verbal memory (standard score)	67	74	46*
Visual memory (standard score)	69	81	59*
LP I (%ile)	5th	15th	2nd
LP II (%ile)	<1st	<1st	<1st
VR I (%ile)	13th	19th	–
VR II (%ile)	<1st	<1st	–
**WRMT (/50)**			
Words	26	21	–
Faces	25	25	–
**CVLT**			
Acquisition (*T*-score)	12	9	38
Short delay free (*Z*-score)	−4	−4	−4
Long delay free (*Z*-score)	−4	−4	−3
Recog. discrim. (*Z*-score)	−3	−4	−2
**ROCF (/36)**			
Copy	36	35	36
Immediate recall	4	–	<20 (*Z*-score)
Delayed recall	0	0	<20 (*Z*-score)
**AMI Autobiographical (/9)**			
Childhood	2	7	–
Early adult life	3	6	–
Recent life	1	3	–
**AMI personal semantics (/21)**			
Childhood	16	17.5	–
Early adult life	13.5	21	–
Recent life	8	16	–
Judgment of line orientation (/30)	23	26	24
Benton visual discrimination test (%ile)	>95th	–	–
Benton face recognition test (%ile)	1st	–	33rd–59th
Letter fluency^3^ (scaled score)	6	8	11–12
WAIS-R digits (scaled score)	12	13	–
**WCST**			
Categories (/6)	–	6	6
Persev. resp. (*Z*-score)	–	−0.5	–

*WAIS-R, Wechsler Adult Intelligence Scale – Revised; AM-NART, American National Adult Reading Test; WMS-R, Wechsler Memory Scale – Revised; LP, logical passages; VR, visual reproduction; CVLT, California Verbal Learning Test; ROCF, Rey Osterrieth complex figure; AMI, Autobiographical memory interview; WCST, Wisconsin Card Sorting Test*.

*^1^Number in parentheses represents standard score on the Wechsler Abbreviated Scale of Intelligence (WASI) from a 2003 re-assessment of general intellectual function*.

*^2^Score is based on the number of animal names produced in 1 min*.

*^3^Score is based on the total number of words produced for the letters F, A, and S when given 1 min for each*.

*WMS-III (Hurley et al., 2011).

To ensure that the patients’ memory problems would not confound their answers on our questionnaire, we administered it to patients’ family members: K.C.’s mother, D.A.’s wife, and H.C.’s mother and fiancé. We used the ratings from K.C.’s mother and D.A.’s wife, and from H.C. herself (we corroborated H.C.’s answers with her mother’s and fiancé’s, and found that their answers were similar to hers). Although these family members know the patients very well and the patients’ interactions with others are relatively limited (making us confident that their answers on behalf of the patients were *reasonably* accurate), we acknowledge that it is possible that these informants are not fully aware of the individuals with whom the patients may discuss problems or concerns.

Twenty healthy older men (age *M* = 66 years, range = 57–72 years; education *M* = 17 years; range = 13–23 years) served as controls for K.C. and D.A. Eighteen healthy young women (age *M* = 20 years, range = 17–26 years; education *M* = 13 years; range = 12–16 years) served as controls for H.C.

### Questionnaire and procedure

We administered the National Social Life, Health, and Aging Project’s Social Network Module (Cornwell et al., 2009) to close relatives of each amnesic patient (see Participants), to patient H.C. herself, and to controls. This questionnaire assesses social network size and composition by collecting egocentric network data: the respondent identifies a set of people in his or her network and comments on the emotional closeness and connectedness of the relationship(s) linking them. The questionnaire uses *name generator* questions to prompt participants to enumerate relevant family and friends. With this method, it is possible to identify several different types of network members: core confidantes (Roster A), other potentially important network members (Rosters B and C), and any remaining household members (Roster D). We combined these four rosters in our analyses of social network size and density.

In keeping with the standard procedure, we introduced the questionnaire with: “From time to time, most people discuss things that are important to them with others. For example, these may include good or bad things that happen to you, problems you are having, or important concerns you may have. Looking back over the last 12 months, who are the people with whom you most often discussed things that were important to you?” If participants did not give an answer, we prompted them with: “This could be a person you tend to talk to about things that are important to you.” After participants finished naming an initial group of close family and friends in Roster A, we asked: “Is there anyone else?” Any names listed after this prompt were included in Roster B. Then we asked: “Is there anyone else that you haven’t named, perhaps someone with whom you feel especially close?” Any additional names were added to Roster C. Finally, we asked: “Is there anyone that you haven’t named yet that lives in the same house as you?” Any names elicited at this point were added to Roster D. We altered the standard administration in only one way: whereas the standard procedure involves eliciting details regarding a maximum of five names, we asked for details on as many names as each participant was able to provide.

For each named person, participants specified (1) their relationship (e.g., spouse, mother, neighbor, etc.), (2) their age and gender, (3) whether they live in the same household, (4) for how long they’ve known each other, (5) how often they speak (on an eight-point scale ranging from 1 = *daily* to 8 = being *less than once a year*), (6) how close they are (on a four-point scale ranging from 1 = *not very close* to 4 = *extremely close*), and (7) their likelihood of sharing important concerns (on a three-point scale ranging from 1 = *very likely* to 3 = *not very likely*). Given the lack of sensitivity in this last scale we did not analyze it.

**Figure 1:**
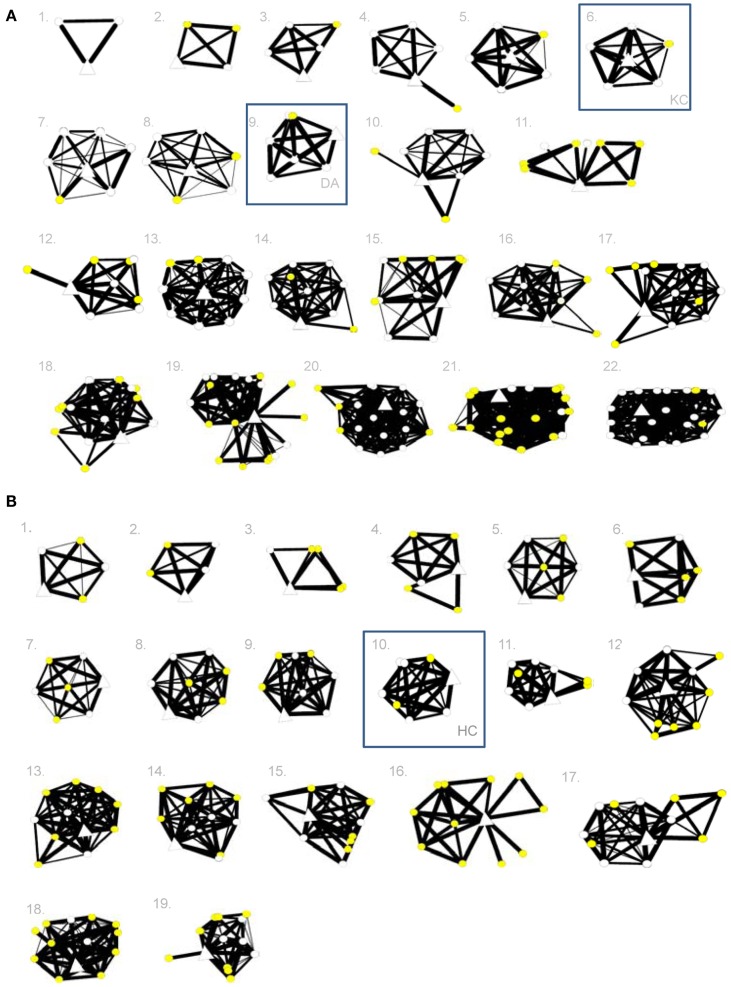
**(A,B)** Social network maps for amnesic patients K.C. and D.A. **(A)** and H.C. **(B)** and their individual controls. In each person’s network, the open triangle represents the individual participant, open circles represent family members, and yellow circles represent friends. A higher degree of relationship “closeness” between two nodes is shown by a thicker line between them. Individuals’ maps are presented in ascending order from less dense to more dense social networks.

In order to obtain information on the density of their networks, we asked participants to specify the frequency of contact between each possible pairing among the identified family and friends (with the highest frequency being *daily* and the lowest frequency being *never*). If participants asked, they were told that communications via the internet or telephone were to be counted. We entered these data into a social network plotting program, Social Networks Visualizer (http://socnetv.sourceforge.net), to create social network maps for each individual, shown in Figure 1.

Finally, participants rated how frequently (on a seven-point scale ranging from 1 = *several times a week* to 7 = *never*) they have engaged in social activities (visiting neighbors, volunteering, and attending religious or social groups) in the past year. Because these four social activity scores were positively correlated across individuals, we combined them into a composite. For all questions, participants could also select “*don’t know*” or “*refuse to answer*” as response options, although everyone used the scales without difficulty.

All participants provided informed consent before beginning, and the project was approved by the Research Ethics Boards of the University of Ottawa, York University, and Baycrest.

## Results

We performed one-tailed single case *t*-tests to compare each amnesic patient to his or her control group (Crawford and Howell, 1998). Given the similarities between patients K.C. and D.A. (in terms of both their demographic characteristics and their data), we present their results together first, followed by results for patient H.C.

### Patients K.C. and D.A.

Although K.C. and D.A. do get out for weekly routine social activities through local brain injury programs (K.C. swims and plays pool, and D.A. plays poker), both were reported to be much less involved in social activities than normal (each scoring 7 on the aggregate social activity scale from 1 = *several times a week* to 7 = *never*; control *M* = 3.43, SD = 1.29, range = 1.25–6; *t*s = 2.70, *p*s = 0.007). Closer to home, both K.C. and D.A. have a normal number of close relationships (K.C. = 5 and D.A. = 6; control *M* = 9.70, SD = 5.69, range = 2–22; *t*s < 1), as shown in Figure 1A. The median closeness of these relationships, estimated using a scale from 1 = *not very close* to 4 = *extremely close*, is normal for K.C. (score = 3; control *M* = 2.98, SD = 0.64, range = 2–4; *t* < 1), but marginally reduced in D.A. (score = 2, *t* = 1.49, *p* = 0.08). The median amount of contact between these patients and their close others, using a scale from 1 = *every day* to 8 = *less than once a year*, is comparable to controls for both patients (K.C. = 4 and D.A. = 3; control *M* = 2.95, SD = 1.21, range = 1–5; *t*s < 1).

K.C.’s and D.A.’s relationships tend to have been established pre-morbidly and to stay within the family. For example, K.C. has made no new close friends in the three decades since the onset of his amnesia, and D.A. has made just one new friend in the two decades since his. This is below average (for K.C., control number of friends made since 1981 *M* = 5.11, SD = 4.21, range = 0–16; *t* = 1.18; for D.A., control number of friends made since 1993 *M* = 3.42, SD = 4.02, range = 0–15; *t* < 1), albeit not significantly. A somewhat higher-than-normal percentage of their relationships are with nuclear family members (K.C. = 80% and D.A. = 83%; control *M* = 49%, SD = 25%, range = 13–83%; for K.C., *t* = 1.21, *p* = 0.12; for D.A., *t* = 1.33, *p* = 0.10). The density of K.C.’s social network (i.e., the ratio of existing links to all possible links) is a little higher than average, and D.A.’s is a little lower than average, but both are within the normal range (K.C. = 1.00 and D.A. = 0.48; control *M* = 0.70, SD = 0.23, range = 0.30–1.00; for K.C., *t* = 1.27, *p* = 0.11; for D.A., *t* < 1). In the past, K.C. has volunteered at a local library and participated in outpatient programs, including organized social outings to a community center. Now, however, other than an annual visit from an old friend and the occasional outing, his social interactions have become more limited, to his parents, siblings, and his parents’ friends. After his encephalitis, D.A. used to volunteer at a school and community group, but he has recently ceased both these activities and now focuses on visiting occasionally with family and friends.

### Patient H.C.

Throughout her life, H.C. has been provided with opportunities to make and keep up social links: she attended regular schooling, and uses her smartphone for organizing her social life and her computer for social networking. Yet, she and her family still estimate that she is less involved in social activities than normal (7 on a scale from 1 to 7; control *M* = 4.53, SD = 1.44, range = 1.5–6.5; *t* = 1.67, *p* = 0.06).

Despite this, and despite her lifelong memory impairment, H.C. has established a normal number of close relationships (7; control *M* = 8.06, SD = 2.71, range = 4–12; *t* < 1). Two of these relationships are with people outside of her family, which is within the range of normal (control *M* = 4.89; SD = 2.68, range = 2–11; *t* = 1.05). One of these relationships is particularly notable: she is engaged to be married. The estimated closeness of her relationships is normal, with a median score of 4 on a scale from 1 to 7 (control *M* = 3.31, SD = 0.55, range = 2–4; *t* = 1.22), and so is her degree of contact with these people (3 on a scale from 1 = *every day* to 8 = *less than once a year*; control *M* = 2.08, SD = 1.05, range = 1–5; *t* < 1). The density of her network is normal (0.75; control *M* = 0.73, SD = 0.23, range = 0.36–1.00; *t* < 1) H.C. spends most of her time with her family, her fiancé, and a small group of friends from high school. The majority of her socializing takes place at the movies, at her friend’s house, and occasionally at a restaurant or bar, although when she goes out to these places she usually only does so with one person or a small group (i.e., her fiancé, sister, mother, and/or friend). H.C.’s mother describes her lifestyle as becoming increasingly sedentary and routine in recent years.

## Discussion

We examined the potential significance of memory to close interpersonal relationships. Overall, the picture presented by our three amnesic patients is of social networks being winnowed, although not necessarily obliterated, by amnesia. Cases K.C. and D.A. are less involved than normal with neighbors and religious and community groups. The confluence of quantitative and qualitative data suggests that K.C.’s and D.A.’s close relationships tend to be with family members. K.C. has made no new close friends in the 30 years since the onset of his amnesia, and D.A. has made just one in the 20 years since the onset of his. Like the adult-onset cases, H.C. is involved little with neighbors and religious and community groups. On the other hand, she has perhaps been more socially successful than our two adult-onset cases: despite being memory-impaired all her life, she has forged a couple of close relationships outside of her family, including one with her fiancé. This is a notable accomplishment. A marriage is perhaps the most difficult kind of interpersonal relationship for *anyone* to establish and to maintain, let alone someone with a severe memory impairment.

The reduced but not eliminated social functioning exhibited by our patients, along with the potential subtle differences from patient to patient, are consistent with the literature: after patient H. M. underwent his bilateral temporal lobectomy in 1953, his friends appear to have fallen away. He lived with various family members, and after they could no longer care for him he ended up in a nursing home. Nonetheless, even in his later years he was still capable of establishing what might best be described as quasi-friendships, in which he and certain other residents preferred each others’ company (Corkin, 1984; Suzanne Corkin, personal communication, March 23, 2012). Another well-known case, N. A., lost his friends after the onset of his amnesia, but remained close to his mother, learned to get along with her new romantic partner, and became popular at the day treatment center he attended (Kaushall et al., 1981). The amnesic patient Clive Wearing may be the most socially isolated case. A newspaper report on him and his wife Deborah notes that they:

live in a closed, insular world of two. Clive has no friends for the simple reason that he would forget who they are. “We don’t mix,” explains Deborah. “Clive lives in his unit and goes out accompanied by members of staff. Occasionally when he’s out with me he will say strange things to people in cafes like, ‘Are you the Prime Minister?,’ [or] ‘Are you the Queen of England?’ It’s because they are the first person he has seen since waking from ‘unconsciousness’ that minute, so they must, he presumes, be important.” (France, 1995)

Taken together, our data and the descriptions of these other amnesic patients (see also Tate, 2002; Gupta et al., 2009; Warren et al., 2012; but see Wilson, 1999; Duff et al., 2008) suggest that episodic memory may serve as a kind of “social glue,” enabling people to form social bonds more rapidly and easily, and to maintain them over the years. Even with considerable effort from family members, many amnesic patients get out less often than normal, and participate less often in community events and activities, giving them fewer opportunities to make new friends (see text footnote 1). Even if they do meet new people, the inability to consciously recollect those people, their names, the places and times they have met, and what happened (and difficulty using these memories flexibly and updating them when necessary) likely further hampers social functioning. Consistent with this idea, an initial report suggests that in healthy people episodic memory abilities predict social network size (Stiller and Dunbar, 2007). In fact, Dunbar has argued that our social networks have an upper limit in size (“Dunbar’s number”; Dunbar, 1992) partly because we can only recollect in detail a certain number of people. However, what is intriguing is that not all amnesic patients appear to be hindered to the same degree (e.g., Wilson, 1999; Duff et al., 2008). We consider potentially important factors below.

### Factors to consider in future work

The accumulating data on episodic memory and social functioning are intriguing, and set the stage for further research. However, several other factors might help to explain the variability among amnesic patients (and among healthy people, for that matter) and should be included explicitly in future work, including:

#### Developmental and sex differences

Might developmental amnesic patients (such as case H.C., described here) be more socially resilient than adult-onset patients? If so, it could be due to their memory and other cognitive impairments often being less severe than those of adult-onset cases (Cooper et al., 2011). Another possibility is that developmental amnesic patients’ family and friends have never known them to be different, and are not required to adjust to the sudden appearance of a memory impairment. It is interesting to note that, like H.C., many of the adult patients with developmental amnesia studied by Faraneh Vargha-Khadem and colleagues are involved in serious relationships (personal communication, August 17, 2012). A formal comparison of adult-onset and developmental cases would be informative.

It might also be that women are better able than men to deal with the consequences of memory impairment. Warren et al. (2012; see also Duff et al., 2008) raised this intriguing possibility in discussing two of their more socially successful amnesic patients (cases 1846 and Angie), who, like our case H.C., are women.

#### Emotional functioning

Close relationships require us to experience, understand, communicate, and occasionally control our own emotions, and to interpret others’ and then behave appropriately. Two brain regions particularly crucial to these functions are the frontal cortex and the amygdala. Might amnesic patients’ social problems be attributable to concomitant frontal and/or amygdalar damage? Among our patients, K.C. and D.A. do have amygdalar involvement and D.A. has orbitofrontal damage too (Rosenbaum et al., 2005, 2008), whereas, as far as we can tell, H.C. does not. The literature is mixed, though: on the one hand, structural neuroimaging studies of these regions in healthy people suggest that smaller volumes are associated with smaller and/or less complex social networks (Powell et al., 2010, 2012; Bickart et al., 2011; Lewis et al., 2011; Kanai et al., 2012). On the other hand, some patients with damage to these regions show only subtle, if any, impairments. For example, when Becker et al. (2012) examined the social networks of identical twins with focal amygdalar damage, one twin had a smaller than normal network but the other was better connected socially than many controls.

#### Personality and motivation

People who are less neurotic and more extraverted, open, agreeable, and conscientious are more likely to make and keep friends (e.g., Pollet et al., 2011). Amnesic patients vary in their personalities, and, what is more, adult-onset cases’ personalities can subsequently change. For example, among our patients, K.C. was a gregarious thrill-seeker before his brain injury and by all accounts had a large social network. He is quiet and reserved now and undoubtedly has a smaller network. In contrast, developmental case H.C. has always had a vivacious personality, which may be the reason for her relative social success. D.A. falls somewhere in between the other two patients: he has a personality that is similar to H.C.’s but not as much social success (in fact, his social relationships seem to be limited to couples and depend on his wife).

A related question is that of motivation: many of the more socially successful amnesic patients have been described as particularly motivated to recover (and/or to not appear to be impaired). Duff et al.’s (2008) successful case Angie worked hard to conceal her memory impairment from others; Wilson (1999) described two similar cases (Jay and Alex). Among our patients, D.A. appears to be the most similar to Angie in this regard.

#### Opportunities to socialize

No matter how motivated one might be, myriad contextual factors (as varied as family size, neighborhood density and services, available transportation, local crime rates, and family and cultural norms) affect one’s chances of making and keeping up social bonds. Some amnesic patients follow regimented routines and stay close to home. In these cases opportunities to socialize (especially with new people, but also with old friends and family) are limited. In contrast, our most socially successful patient, H.C., had normal schooling and has been steadfastly supported by her family in exploring new venues, including summer camps, part-time jobs, trips to the movies, and social networking on the computer. These opportunities may have been the crucial factor allowing her to forge a few relationships outside of her family. (Note: of course, these opportunities may have been more available to H.C. because of the young age at which she exhibited episodic memory impairment.)

#### Other aspects of memory and cognition

Although all amnesic patients share a profound anterograde episodic memory impairment, the severity of this impairment can vary somewhat from case to case (as can the locus and extent of damage in the brain). At present, we have too few data to know whether the degree of anterograde amnesia is what separates the more socially successful patients from the less successful ones, although the initial data from healthy people would support this prediction (Stiller and Dunbar, 2007). However, other aspects of declarative memory (including retrograde episodic memory, and anterograde and retrograde semantic memory abilities) can also vary from patient to patient and may be important. Might those patients who are more capable of new semantic learning be more successful in forging new friendships? Might those who are able to draw on intact pre-morbid memories be more likely to retain old friendships? Might those who can develop a vague sense of familiarity (despite no recollection) grow more comfortable with someone over repeated encounters with him or her? These are interesting questions for future work, while bearing in mind the potential importance of other cognitive faculties, including problem-solving, theory of mind, executive function, future thinking, processing speed, meta-cognition, attention, language, and overall intelligence.

The variables outlined above (among others) must be taken into account in future research. A multi-factorial study on how memory impairment fits in with these other potential influences would be quite useful. Because individuals with selective episodic memory impairment are so rare, it might be fruitful to also use this approach with other groups with memory problems (e.g., normal aging). Another potentially productive avenue is experimental work with animal models, in which each of the potentially important factors outlined above can be better-controlled. Even though non-human primate social interactions are not as sophisticated as human ones, it is possible to make discrete lesions to particular brain areas, and to control the animals’ social contexts (e.g., Bauman et al., 2004, 2006; Machado and Bachevalier, 2006; Goursaud and Bachevalier, 2007; Machado et al., 2009; Toscano et al., 2009).

### Other future questions

A useful future project would be to differentiate pre-morbid social networks from post-morbid ones. As mentioned above, K.C. was the “life of the party” before his amnesia, but now has relatively few social links. How do social networks *change* following the onset of amnesia? K.C.’s and D.A.’s family members were willing to try to reconstruct their pre-morbid social lives from decades ago, but when we explored this idea with controls it became apparent that it would be very difficult to obtain reliable data with this method. A more informative approach might be to track how recent-onset cases’ social networks change longitudinally after the onset of amnesia. Other future methods might be to verify with nominated friends that their feelings are mutual, and to probe what these friends see as the strengths and deficiencies of the patients.

In future work, incorporating other kinds of patients would be beneficial. First, it would be useful to know what the similarities and differences are between amnesia and other severe cognitive deficits (for example, aphasia: Davidson et al., 2008; Northcott and Hilari, 2011). Second, such work might help us learn the degree to which the mere *stigma* associated with brain injury and cognitive deficits might hamper social bonding. That is, in comparison to other patients with similarly limited opportunities for social engagement and who have been labeled as “brain damaged” or “mentally impaired,” are memory-impaired people less likely to establish and maintain good social links? (See text footnote 1). Finally, in the present study, we did not explore patients’ subjective feelings. Are they lonely? In the past, one of us (R. S. R.) has asked K.C. about this, and he has responded “no.” A report from Gupta et al. (2009) suggests that other amnesic patients might be lonely (depending on their degree of insight into their memory impairments), and a recent study of older adults with amnestic-type Mild Cognitive Impairment (Parikh et al., 2012) suggests that this group too experience feelings of loneliness and isolation.

Our social lives are inherently complex, and memory may be more important for establishing and maintaining some kinds of relationships than others. Whatever close relationships remain in amnesic patients are often familial, in which the bonds are supported by kinship or social norms, such as marriage vows. Indeed, one of the most poignant consequences of memory impairment is the heavy weight it places on these family ties. It is heartening that so often, under such tremendous strain, these ties bend but do not break.

## Conflict of Interest Statement

The authors declare that the research was conducted in the absence of any commercial or financial relationships that could be construed as a potential conflict of interest.

## Footnotes

^1^We are grateful to an anonymous reviewer for this suggestion.
